# Pepino Mosaic Virus in Tomato: Challenges, Control Strategies, and Future Prospects for Resistance Breeding

**DOI:** 10.3390/ijms262311749

**Published:** 2025-12-04

**Authors:** Marzena Nowakowska, Julia Minicka, Marcin Nowicki, Wojciech Szczechura, Beata Hasiów-Jaroszewska

**Affiliations:** 1 Department of Genetics, Breeding, and Biotechnology of Vegetable Crops, the National Institute of Horticultural Research, 96-100 Skierniewice, Poland; wojciech.szczechura@inhort.pl; 2 Department of Virology and Bacteriology, Institute of Plant Protection-National Research Institute, 60-318 Poznań, Poland; j.minicka@iorpib.poznan.pl; 3 Department of Entomology and Plant Pathology, Institute of Agriculture, University of Tennessee, Knoxville, TN 37996, USA; mnowicki@utk.edu

**Keywords:** cross-protection, genome editing, PepMV, plant–pathogen interactions, resistance breeding, RNA silencing, tomato virology

## Abstract

Pepino mosaic virus (PepMV) is a highly infectious potexvirus that poses a significant threat to tomato cultivation in greenhouses worldwide. The threat posed by this virus is attributed to by its genetic complexity, characterized by the presence of multiple genotypes in circulation, mixed infections, and ongoing genotype turnover. Surveys of wild *Solanum* species have identified promising sources of resistance; however, this resistance is often incomplete, manifesting as symptomless, yet virus-positive, plants. When resistance is identified, introgressing of these traits into elite backgrounds is frequently impeded by reproductive barriers and linkage drag. Consequently, there are currently no commercially available cultivars with durable resistance to PepMV. Current control measures rely on stringent hygiene practices, seed health protocols, and the use of mild isolate cross-protection, which can mitigate fruit symptoms when carefully genotype-matched and closely monitored. Looking forward, achieving durable control will likely require host-centered strategies. Loss-of-susceptibility mutations and RNA interference-based approaches have demonstrated strong potential in experimental studies. Future solutions may involve the integration of genome editing with RNA-based technologies, supported by regulatory harmonization and socioeconomic viability considerations.

## 1. Introduction

Pepino mosaic virus (genus *Potexvirus*, family Alphaflexiviridae, species *Potexvirus pepini*) has emerged as a major challenge in tomato (*Solanum lycopersicum*) cultivation worldwide [1,2]. Its rapid spread, mechanical transmission, and formidable genetic plasticity, marked by rapid molecular evolution, mixed infections, and frequent genotype shifts, pose substantial challenges to sustainable control measures [3,4,5,6,7,8,9]. Currently, the most effective strategy for minimizing losses involves cross-protection using mild isolates in conjunction with stringent hygiene and seed sanitation practices [10,11,12]. This biologically based approach has markedly reduced fruit marbling and yield decline in commercial crops and remains the cornerstone of PepMV management in tomato plants. However, it does not eradicate the virus, and its success is contingent on continuous monitoring and meticulous implementation. Despite extensive research efforts, no tomato cultivar with stable genetic resistance to PepMV has yet been developed.

Understanding why durable resistance is lacking and how it can be achieved has become a pivotal question in tomato virology and tomato breeding. This review focuses on the biological and molecular factors contributing to this issue and explores emerging strategies, such as tolerance breeding, RNA-based methods, and genome editing, which may aid in the development of more durable resistance to PepMV in tomato.

## 2. Biology and Diversity of PepMV

### 2.1. General Characteristics, Host Range, and Transmission

PepMV is a positive-sense, single-stranded RNA virus that has expanded from a limited host range in pepino (*S. muricatum*) to a global pathogen of greenhouse tomatoes (*S. lycopersicum*). First identified in Peru in 1974 [2], PepMV remained relatively unknown until the late 1990s, when it suddenly appeared in European tomato crops and subsequently spread across North America and other major production areas [1,13,14,15,16,17,18,19,20,21,22].

PepMV primarily circulates in intensive tomato cultivation, although it can infect numerous other Solanaceae species [2,6,22,23,24]. Non-solanaceous hosts, such as basil, garlic, and faba beans, have also been shown to be hosts for PepMV, as well as weeds from various plant families, although their relevance to the epidemiology of PepMV has yet to be determined [1,9,25,26,27,28,29].

PepMV poses a threat to tomato crops because of its highly efficient transmission ability [1,29]. The virus spreads primarily through mechanical contact during routine crop management activities, such as pruning, deleafing, and harvesting. Contaminated hands, tools, and equipment serve as means of transmission, with dense canopies and intensive labor practices exacerbating this risk. Seed and transplant pathways are particularly critical for long-distance spread, as PepMV can be present in commercial seed lots and can introduce latent infections into nursery plants [30]. Although the seed-to-seedling transmission efficiency is low (0.005–2%) and the virus is restricted to the seed coat rather than the embryo, the global scale of seed trade makes even minimal rates epidemiologically significant [6,9,30,31,32]. The trading of infected tomato fruits is a crucial pathway for long-distance dissemination. A study in northern Europe found PepMV in over 60% of the tested imported tomato batches, demonstrating that these fruits can serve as a potent source for introducing the virus into new areas [29]. Mechanical inoculation tests confirmed that the viral isolates from these fruits were easily transmitted to healthy tomato plants.

Within cultivation facilities, PepMV can rapidly disseminate through grafting processes and recirculating irrigation systems [15]. Although bumble bees, soil fungi, and whiteflies (*Trialeurodes vaporariorum*) may act as mechanical carriers [33,34,35,36,37,38], no persistent insect transmission has been demonstrated. The use of parasitoids for pest control has been reported to have a neutral effect on PepMV transmission [39]; however, zoophytophagous predators, such as *Nesidiocoris tenuis*, can complicate integrated pest management strategies owing to their potential for fruit injury and interactions with virus-infected crops [40]. The persistence of the virus in soil, plant debris, and irrigation water is not high [41], especially in comparison with other highly persistent viruses, such as tobamoviruses. Nonetheless, mixed infections, including those identified in imported fruit, may involve either different PepMV genotypes or co-infecting viruses (e.g., *Tobamovirus fructirugosum*, tomato brown rugose fruit virus, ToBRFV). Such interactions may create conditions conducive to recombination or synergism [6,9,42,43]. Although recombination between PepMV genotypes offers opportunities for viral recombination and the emergence of novel variants with potentially increased aggressiveness, heterologous mixed infections with unrelated viruses have also been demonstrated to enhance symptom severity, occasionally mimicking those of aggressive PepMV genotypes. Furthermore, plants may recover symptomatically while remaining infected, creating cryptic reservoirs that sustain viral spread and complicate control efforts [1,9,15].

### 2.2. Symptomatology

**Figure 1:**
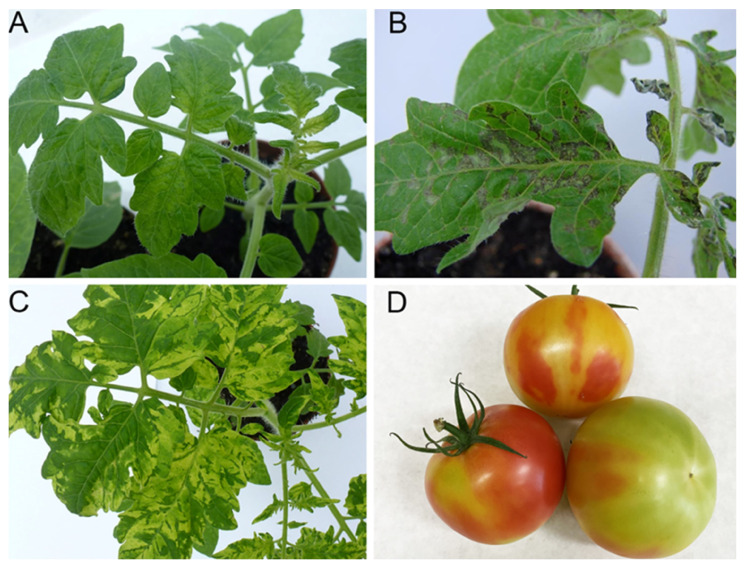
Different types of symptoms caused by PepMV: (**A**) mild mosaic; (**B**) leaf necrosis, (**C**) yellowing, (**D**) fruit marbling. Original image prepared by the authors.

PepMV symptoms in tomato are highly variable and can occur with or without foliar signs, affecting leaves, stems, and, most critically, fruits. Foliar symptoms include mild, light-green (Figure 1A) to bright-yellow mosaics (Figure 1C), blistering/bubbling and other leaf distortions, chlorosis, occasional necrosis (Figure 1B), green stem striations, and overall growth reduction [1,44]. Fruit symptoms, often the main cause of commercial loss, comprise mottling and blotchy, irregular pigmentation (Figure 1D) with occasional necrosis, and may appear even when the foliage is symptomless [1,44]. Symptom severity depends on multiple factors, notably the viral determinants (genotype, mutation), host background, environmental conditions, and infection status (single vs. mixed infections) [7,45,46,47,48]. Although identifying genetic determinants is challenging, specific point mutations in the PepMV genome have been associated with yellow mosaic or necrosis phenotypes [45,49,50].

### 2.3. Genome Organization and Functional Modules

**Figure 2:**
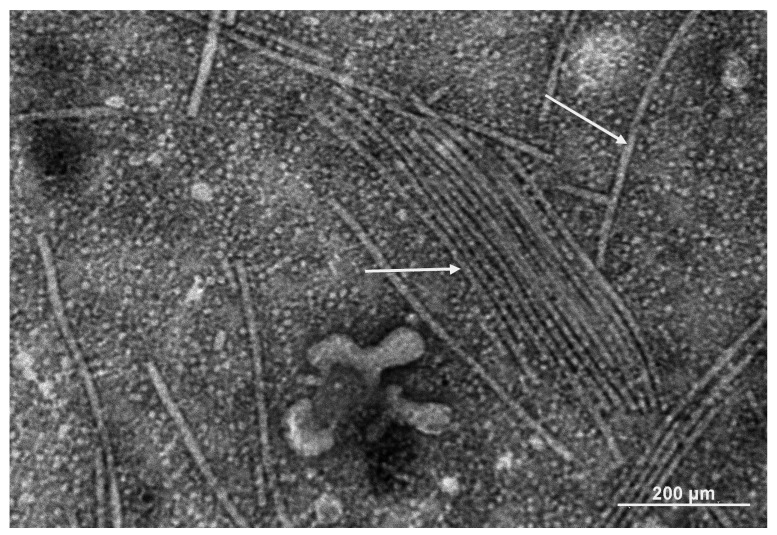
Filamentous PepMV particles (arrows) in transmission electron microscopy (TEM). Bar = 200 µm. Original image prepared by the authors.

PepMV contains a positive-sense, single-stranded RNA molecule approximately 6.4 kb long and is enclosed within a flexible, filamentous, non-enveloped virion (Figure 2) [9,16,48,51]. Within the linear genome, five open reading frames (ORFs) are located between the 5′-m^7^G cap and the 3′-poly(A) tail [13,16]. ORF1 encodes an RNA-dependent RNA polymerase (RdRp, 164 kDa) that includes three conserved regions: a methyltransferase, an NTPase/helicase domain containing NTP-binding motifs, and the polymerase domain. The partially overlapping ORFs 2–4 encode the triple gene block (TGB) proteins (TGB1, TGB2, and TGB3 of 26, 14, and 9 kDa, respectively), which form the conserved movement module of potexviruses. TGB1 is an RNA-binding protein and the primary RNA-silencing suppressor [52], whereas TGB2 and TGB3 target endomembranes and recruit TGB1 to the plasmodesmata [53]. ORF5 encodes a multifunctional coat protein (CP, 25 kDa). The cryoEM structure of PepMV CP in virions revealed three main regions: a flexible N-terminal arm enabling lateral CP–CP contacts, a predominantly α-helical core harboring the RNA-binding pocket, and a C-terminal extension that mediates longitudinal CP–CP interactions, shaping the central channel of the virion [51]. Beyond its structure, PepMV CP is required for cell-to-cell movement of the virus and acts as an RNA silencing suppressor [51,54,55,56]. Additionally, specific point mutations in the PepMV genome have been linked to the loss of potato *Rx* gene recognition [57,58] or influence virus symptoms [44,45], with some mutations located within the CP-coding gene [49,59]. Collectively, PepMV proteins are highly multifunctional in integrating replication, movement, and silencing-suppression activities, providing substantial plasticity and facilitating rapid adaptation to host defense responses.

### 2.4. Genetic Diversity and Evolutionary Dynamics

PepMV demonstrates marked genetic diversity, with isolates categorized into six genotypes based on phylogenetic and whole-genome analyses: Peruvian-like (LP), European (EU), North American (US1/CH1), Chilean 2 (CH2), southern Peruvian (PES), and the newly identified Europe-Asia-Pacific (EAP) lineage [9,13,60]. Phylogenetically, the LP and EU genotypes are closely related, as are the US1/CH1 and PES genotypes, which form a common cluster. In contrast, the CH2 lineage and its derived EAP variant constitute a more distant outgroup [9,60]. Isolates classified as belonging to the same genotype generally exhibited nucleotide identities exceeding 95%. In contrast, the identity between different genotypes varies from about 78% to 94%, which indicates notable evolutionary diversification within the species. Among these, the CH2 and EU isolates are the most distinct, with a sequence identity of roughly 78% [13,48].

Epidemiologically, the EU and CH2 genotypes are dominant in commercial tomato production. Initially, EU isolates spread across European greenhouses but were progressively displaced by the CH2 genotype, which now predominates in several regions [6,50,61,62,63]. The EAP lineage, which is distinct from CH2, has recently been detected in both Europe and the Asia-Pacific region, suggesting the recent emergence and rapid spread [9]. Molecular epidemiology studies support this hypothesis. A major dissemination hub for its global spread may have formed in Chile via the tomato seed trade [9]. The long-term co-circulation of CH2 and EU genotypes has also been documented in Spain [63,64]. Notably, the CH2 and EAP genotypes exhibit codon usage patterns that are more divergent from those of their host, *S. lycopersicum*, compared to other genotypes. This divergence may provide them with an advantage in mixed infections and contribute to their global success [9]. At the population level, CH2 exhibits greater genetic variability than the EU; notably, mixed infections reduce CH2 variability relative to single infections [5]. This contrasts with earlier observations [65], which showed that temperature acts as an ecological driver influencing virus–virus interactions, directly affecting the genetic diversity of co-infecting viruses and, under elevated conditions, enhancing CH2 diversity.

The PepMV population structure is highly dynamic. Mixed infections involving multiple genotypes are common in greenhouse crops. Such infections can modulate viral evolutionary dynamics and constitute a prerequisite for recombination [5,6,29,43,50,63,64]. At least nine chimeric genomes have been described, including a rare case of probable host-to-virus horizontal gene transfer. In this event, an isolate named Ca1A/Canada/MN549397 acquired a 35-nt segment from the host into its 5′ untranslated region (5′UTR) [9]. Notably, the majority of US1 isolates share a shorter 19-nt host-like segment, which may have predisposed or facilitated this type of recombination. Despite an estimated evolutionary rate ranging from moderate to high (6.58 × 10^−4^–5.57 × 10^−3^ substitutions/site/year) and high within-genotype homogeneity, the population structure is further shaped by genetic bottlenecks during systemic movement and eradication/re-emergence cycles [3,6,7,9]. These features exemplify the quasispecies nature of PepMV, in which elevated substitution rates, recombination, and mixed infections fuel rapid adaptation to changing environmental conditions [1,7,8,9,65,66].

In addition to genotype-level diversity, PepMV frequently co-infects tomato plants with other viruses, further complicating disease dynamics. Notably, mixed infections with ToBRFV have been reported at a high incidence [42,67,68]. These co-infections may lead to synergistic effects, such as enhanced symptom severity or altered viral accumulation patterns, although their precise outcomes remain unexplored.

### 2.5. Genetic Determinants of Symptom Expression

Multiple viral proteins contribute to the modulation of symptom severity in PepMV-infected plants. Amino acid changes in the CP, including E155K and D166G in the CH2 genotype modulate leaf phenotypes, such as interveinal yellowing [49]. A distinct amino acid change, E236K, identified in the CP of an aggressive PepMV-EU isolate, induced bright yellow mosaic symptoms in *Nicotiana benthamiana* [48]. Structural modeling places this non-conservative substitution at the penultimate residue of the CP C-terminal extension, a solvent-exposed region that protrudes from the protein core. Its position and the associated charge shift indicate a potential role in modulating interactions with host proteins. Aggressive isolates carrying such mutations, including PepMV-H30 (EU) and PepMV-KLP2 (CH2), are valuable tools for resistance breeding [48], as their well-defined and reproducible phenotypes facilitate the evaluation of host responses.

Genetic determinants of symptom expression also reside outside the CP. In the movement module, a single amino acid substitution of a lysine (K) into glutamic acid (E) at amino acid position 67 of the TGB3 protein (K67E) plays a role in converting a mild pathotype into a necrotic one independently of the genotype used [44,69]. This substitution enhances viral accumulation but is not sufficient on its own to trigger necrosis, which indicates that symptom severity results from the interplay between multiple viral and host factors. Subsequent studies revealed that the mechanism of necrosis induction is more complex than initially assumed. Specifically, the POL domain of RdRp functions as a potent, dose-dependent necrosis elicitor localized within the conserved palm subdomain containing the GDD motif [45]. POL overexpression alone triggers systemic necrosis resembling a hypersensitive response (HR), which is dependent on jasmonic acid (JA) but independent of salicylic acid (SA) signaling. Co-expression of TGB3 and POL domain leads to enhanced necrosis, suggesting that both proteins act synergistically to promote cell death. Environmental conditions further modulate these effects; systemic collapse is most pronounced at moderate temperatures (approximately 26 °C), which confirms that necrosis development is both temperature- and host-dependent [45]. This outcome is accompanied by characteristic biochemical and transcriptomic signatures, including the induction of oxylipin biosynthesis (*9-LOX*, *α-DOX1*), increased reactive oxygen species, and activation of defense-related transcription factors [45].

Host resistance contributes to this additional axis. The potato *Rx* gene (CC-NBS-LRR) confers strong resistance to potato virus X (species *Potexvirus ecspotati*, PVX) [70,71], but only unstable resistance to PepMV when expressed in transgenic tomatoes, with emerging resistance-breaking variants that cause severe *Rx*-mediated necrotic symptoms absent from wild-type plants [57]. This recognition is mediated by PepMV CP, specifically a minimal elicitor region between amino acids 30 and 136, which the study suggests is recognized based on a conserved 3D structure rather than a linear sequence. Resistance-breaking substitutions, A78T, A100D, and A114T cluster within this domain and abolish or severely reduce recognition while still allowing systemic infection, demonstrating a more flexible adaptive mechanism for PepMV with a lower fitness cost than that of PVX [58]. The ability of these mutations to break *Rx*-mediated resistance varies among solanaceous hosts, reflecting species-specific determinants of resistance activation. In *N. benthamiana*, a single amino acid substitution, A78T, is sufficient to evade *Rx* recognition. In contrast, the A114T substitution requires an additional compensatory mutation (A100D) to overcome resistance in tomatoes effectively. In *N*. *tabacum*, however, neither the A78T nor the A114T mutant variant could escape Rx recognition, indicating a more robust *Rx* response in this host [58]. Phenotypically, resistance-breaking events trigger vascular necrosis in *N. benthamiana* and trailing necrosis in tomatoes, illustrating the interplay between viral genotype and host physiology.

Collectively, PepMV disease severity is a network property arising from interactions among CP, TGB3, and RdRp POL domain functions, host immunity (including the *Rx* pathway), and temperature, which has direct implications for breeding strategies (e.g., gene pyramiding) and routine population monitoring of field and greenhouse PepMV isolates.

## 3. Molecular Mechanisms of PepMV–Tomato Interactions

The success of PepMV infection depends not only on the intrinsic properties of its viral proteins but also on the ability of the virus to exploit the host cellular machinery. The virus manipulates the metabolic, redox, and regulatory pathways of tomato to promote its replication, movement, and suppression of host defense mechanisms. As a result of long-term adaptation, PepMV has developed a network of molecular mechanisms that integrates viral and host factors into a finely tuned, infectious system.

### 3.1. Codon Adaptation and Recombination as Evolutionary Drivers

Molecular evolutionary studies have shown that PepMV has adapted to its tomato host at the translational level. Codon usage analyses have revealed that, although PepMV is generally similar to its host codon preferences, certain compositional biases persist [9]. In particular, PepMV has a strong bias against codons containing CpG and favors codons ending with CpA or CpU, a pattern also observed in the host. This is further reflected in the dinucleotide composition, where CpG dinucleotides are under-represented, whereas UpG and CpA are over-represented at the (3,1) codon position, suggesting that the CpG deamination contributes to an uneven nucleotide composition [9]. In contrast, the reduced frequency of UpA appears to result mainly from codon usage bias related to tRNA availability rather than from selection for higher mRNA stability. Such dinucleotide suppression, especially of CpG, may facilitate the evasion of host antiviral surveillance. Interestingly, this adaptation is not uniform across all genotypes. The CH2 and EAP genotypes are less similar to *S. lycopersicum* in codon usage compared to the EU, LP, PES, and US1 genotypes, which might confer a selective advantage in mixed infections and contribute to their global spread [9]. Recombination, including both intergenotypic events and rare host-derived insertions, further contributes to PepMV genomic plasticity [9]. Collectively, codon usage bias, dinucleotide composition, and frequent recombination illustrate how PepMV continuously remodels its genome to optimize replication in the host environment.

### 3.2. Interactions with Host Chaperones

Numerous host proteins have been identified as critical susceptibility factors for PepMV infection, with the heat shock protein (HSP70/Hsc70) family being one of the best-characterized examples. Mathioudakis et al. [72] demonstrated that the PepMV CP specifically binds the tomato Hsc70.3 isoform. Yeast two-hybrid and bimolecular fluorescence complementation (BiFC) assays confirmed this interaction, which localized the complex to the cytoplasm and nucleus. During infection, Hsc70.3 mRNA and protein levels were significantly increased. Crucially, the study demonstrated a direct physical association in infected tissue: Hsc70 was found to co-purify with intact PepMV virions and to co-localize with viral aggregates specifically in the phloem [72]. Based on these findings, the authors proposed that PepMV co-opts Hsc70’s chaperone activity to promote cell-to-cell movement by assisting the translocation of viral ribonucleoprotein (vRNP) complexes through plasmodesmata. Inhibition of Hsp70 activity, either by virus-induced gene silencing (VIGS) or treatment with the flavonoid quercetin, significantly reduced viral accumulation, demonstrating that the Hsp70 machinery is indispensable for systemic movement and replication [55]. Complementary assays in *N. benthamiana* protoplasts showed that the replication of PepMV still depends on HSP70 activity even in the absence of cell-to-cell transport, indicating that these chaperones stabilize replication complexes and may assist in protein folding or virion assembly [55]. Such chaperone-assisted mechanisms typify viral exploitation of host quality-control systems and underscore how PepMV converts general stress-response proteins into specialized infection partners.

### 3.3. Redox Regulation and Host Oxidative Enzymes in PepMV Infection

The redox condition of the PepMV CP plays a crucial role in controlling viral infections. A highly conserved cysteine residue (Cys127) situated within the RNA-binding pocket of CP, can undergo reversible oxidation and S-glutathionylation [56]. These post-translational changes seem to influence CP functions, including its capacity to form virions or virus-like particles (VLPs). In vitro, S-glutathionylation of CP blocks the formation of VLP, which suggests that oxidation inhibits CP self-assembly. This was strikingly confirmed in planta, where a non-oxidizable mutant (CP^C127S^) formed abundant, long VLPs, whereas the wild-type CP produced only short, sparse particles. Although the C127S mutation resulted in a viable virus, its fitness was notably reduced [56]. This discovery provided the first experimental proof that oxidative post-translational modifications can directly influence the activity of a plant viral protein, thereby introducing redox signaling as an additional regulatory layer in plant-virus interactions.

Building on this concept, tomato tau-class glutathione S-transferase SlGSTU38 has been identified as a host factor that specifically interacts with CP [73]. *SlGSTU38*-knockout (gstu38) tomato mutants generated via CRISPR/Cas9 exhibited a cell-autonomous reduction in PepMV accumulation in plants and protoplasts, without altered susceptibility to unrelated viruses such as tobacco mosaic virus (species *Tobamovirus tabaci*, TMV) or PVX, which indicated that SlGSTU38 acts as a PepMV-specific susceptibility factor. SlGSTU38 localizes to PepMV viral replication complexes (VRCs) formed on the endoplasmic reticulum membranes, and its transcript is constitutively more abundant than that of closely related GST isoforms [73]. Loss of SlGSTU38 led to elevated accumulation of reactive oxygen species, enhanced peroxidase activity, and deregulation of stress-responsive genes, suggesting that this enzyme contributes to cellular redox homeostasis. These effects imply that disruption of redox balance may trigger early defense activation, thereby limiting viral replication. Interestingly, *gstu38* plants showed no developmental abnormalities, suggesting that SlGSTU38 specifically contributes to viral compatibility rather than metabolic processes. These insights suggest that PepMV may co-opt a host redox enzyme to support its replication, positioning SlGSTU38 as a potential target for breeding or genome-editing strategies, likely in combination with additional resistance factors, to enhance PepMV management [73].

Beyond CP-mediated processes, PepMV TGBp1 (also called p26) manipulates the host antioxidant systems to suppress defense signaling. TGBp1 directly interacts with tomato catalase 1 (CAT1), a key enzyme responsible for hydrogen peroxide (H_2_O_2_) detoxification [52]. This interaction, confirmed to occur in the cytoplasm and nucleus, leads to the recruitment of CAT1 to viral replication sites (viroplasms). During infection, CAT1 enzymatic activity increases in a dose-dependent manner without a corresponding change in its gene or protein expression, which indicates post-translational activation by p26. Importantly, transient expression of p26 alone was sufficient to enhance catalase activity and reduce intracellular H_2_O_2_ accumulation, thus attenuating ROS-mediated antiviral signaling and favoring viral replication [52]. Silencing of *CAT1* expression via VIGS led to dramatic reductions in PepMV RNA and CP accumulation, demonstrating that CAT1 acts as a positive regulator of infection. Ultimately, the p26-CAT1 interaction serves as a mechanism to suppress host defenses and promote systemic viral infection [52].

In parallel, TGBp1 interacts with the thioredoxin-domain-containing protein, SlTXND9, which possesses an incomplete redox motif (W-PC) and is homologous to phosducin-like protein 3 (PLP3), which is involved in microtubule assembly [74]. SlTXND9 accumulates at the ER-plasmodesmata interface, which is a key site for viral movement, suggesting this protein functions as a scaffold that guides viral ribonucleoprotein (vRNP) complexes along the cytoskeleton. Although its transcript levels remained unchanged, its spatial redistribution upon infection indicated that PepMV exploits constitutively expressed host components to facilitate directed intracellular trafficking [74].

Collectively, these complementary interactions, with SlGSTU38, CAT1, and SlTXND9, illustrate how PepMV integrates host redox regulation and cytoskeletal dynamics to coordinate replication and cell-to-cell movement. By co-opting multiple enzymatic and structural host components, the virus optimizes its replication environment while mitigating oxidative stress, revealing redox control as a central element of PepMV pathogenicity and a promising target for resistance breeding.

### 3.4. Epitranscriptomic Manipulation: m^6^A Methylation and Autophagy

Beyond redox and membrane-associated regulation, PepMV interferes with post-transcriptional control by targeting the N^6^-methyladenosine (m^6^A) RNA modification pathway. In plants, m^6^A is the most abundant internal mRNA modification and plays a pivotal role in regulating transcript stability, splicing, and translation [75,76,77]. The m^6^A writer complex comprises the methyltransferase MTA and the E3 ubiquitin ligase–like protein HAKAI [76]. m^6^A modifications in the PepMV RNA genome are predominantly enriched near the 3′ terminus of the viral genome, a pattern conserved between *S. lycopersicum* and *N. benthamiana* [76,77]. These modifications are part of a mechanism that promotes the degradation of viral RNA, thereby limiting replication. The writer components MTA and HAKAI act as an antiviral barrier; deficiency of these writers facilitates PepMV infection, whereas their overexpression suppresses it [76]. This antiviral activity is linked to the m^6^A modification of viral RNA. YTH-domain readers such as ECT2A, ECT2B, and ECT2C recognize the methylated viral RNA and recruit nonsense-mediated decay (NMD) factors UPF3 and SMG7, promoting the degradation of PepMV transcripts. Through this multilayered mechanism, m^6^A methylation operates as an epitranscriptomic checkpoint that restricts viral proliferation [76].

PepMV utilizes a specific counter-defense mechanism to counter host defenses. Viral RdRp interacts with the N-terminal RING domain of SlHAKAI via the conserved GDD motif within the RdRp2 region and recruits the autophagy regulator SlBeclin1 [77]. This interaction initiates ATG7-dependent autophagic degradation of SlHAKAI, thereby dismantling the m^6^A writer complex and suppressing host methylation-mediated defenses. Inhibition of autophagy, either chemically or by silencing ATG7, prevents SlHAKAI degradation and restores its antiviral activity [77]. The RdRp-Beclin1-HAKAI complex forms distinct cytoplasmic puncta corresponding to active autophagosomes, thus illustrating that PepMV hijacks selective autophagy rather than the ubiquitin–proteasome system to neutralize epitranscriptomic immunity in host cells.

### 3.5. Host Susceptibility Factors: The Role of SlOSCA4.1

Membrane-associated host factors also influence susceptibility to PepMV. The calcium-permeable mechanosensitive channel SlOSCA4.1, an ortholog of *Arabidopsis* AtOSCA4.1, was identified as a key susceptibility factor required for infection [78]. In a large screening of an EMS-mutagenized tomato population, a recessive allele in *SlOSCA4.1* (line 2F531) conferred strong resistance to PepMV. This allele contains a premature stop codon at amino acid position 554, resulting in a truncated protein lacking a significant portion of its C-terminal transmembrane domain. The resistance phenotype was characterized by the absence of symptoms and a marked reduction in viral accumulation across both the EU and CH2 isolates. This resistance was specific to PepMV, as plants remained susceptible to unrelated viruses such as cucumber mosaic virus (species *Cucumovirus CMV*), TMV, and PVX. Independent CRISPR/Cas9 knockout lines confirmed that *SlOSCA4.1* disruption reproduced the resistant phenotype without affecting plant growth or development [78]. Functional assays indicated that SlOSCA4.1 acts early during infection in a cell-autonomous manner. This protein was observed to re-localize from the endoplasmic reticulum to virus-induced replication complexes in infected cells, thus supporting its direct role in facilitating the viral cycle. It has been proposed that SlOSCA4.1 contributes to calcium (Ca^2+^) homeostasis, which is essential for viral replication, a mechanism that resembles the role of calcium signaling in some animal virus infections [78]. Although its *Arabidopsis* ortholog is known to be involved in vacuolar trafficking, experiments in tomato have yielded contradictory results, leaving the role of SlOSCA4.1 in this process to be clarified [78].

The resistance conferred by the *slosca4.1* allele was durable across multiple PepMV genotypes and maintained through serial passages without any detectable growth or developmental penalties. These findings identify SlOSCA4.1 as a novel class of susceptibility factor, distinct from canonical translation initiation factors such as *eIF4E* [78]. SlOSCA4.1 belongs to the OSCA4 clade, an evolutionarily distinct and less-studied branch of the OSCA family, which highlights its potential as a promising and novel target for breeding durable, loss-of-susceptibility (LOS) resistance in tomato.

### 3.6. Integrative Perspective

PepMV infection results from a multilayered interplay between the viral and host components. The virus exploits molecular chaperones (HSP70 family), redox enzymes (GSTU38 and CAT1), thioredoxin-like scaffolds (SlTXND9), and the m^6^A–autophagy axis (SlHAKAI–Beclin1) to sustain efficient replication while suppressing defense responses. These mechanisms, combined with the high evolutionary flexibility of CP, TGB1, and RdRp, enable rapid adaptation to diverse environments and host genotypes. Understanding these processes provides the foundation for identifying durable targets-such as host susceptibility factors-for resistance breeding and genome editing strategies. The molecular mechanisms outlined above directly shape the transcriptomic and physiological reprogramming of tomato during infection, which are discussed in the following section.

## 4. Host Responses and Transcriptomic Reprogramming

PepMV infection elicits complex and dynamic physiological and transcriptional responses in tomato. These include the massive reprogramming of primary metabolism, oxidative balance, hormone signaling, and post-transcriptional gene regulation. Transcriptomic analyses have revealed that infection with PepMV triggers multilayered host responses that vary with the viral genotype, infection stage, and environmental conditions [79,80,81,82,83]. Extensive transcriptional changes occur soon after inoculation with PepMV (4 days post-inoculation, dpi), but the number of responsive transcripts diminishes by 12 dpi, which may be related to the recovery phenomenon commonly observed in PepMV-infected tomato crops [79]. This was further confirmed by Alcaide et al. [80], who found that the number of differentially expressed genes was highest at 7 dpi compared to later time points, indicating that stronger transcriptomic alterations occur at early infection times. The study also noted that although single infections with different genotypes (EU and CH2) showed the most upregulated genes at 7 dpi, this pattern shifted in mixed infections, where more genes were up-regulated at 14 and 21 dpi.

### 4.1. Early Host Reprogramming and Metabolic–Defense Integration During PepMV Infection

PepMV infection triggers profound transcriptomic reprogramming in tomato, which results in redirecting metabolic resources toward stress and defense responses that ultimately fail to confer resistance. The virus disrupts core energy pathways; however, the direction of changes in photosynthesis-related transcripts appears to depend on the context and the infection phase [79,83]. Microarray profiling revealed the predominant repression of photosynthesis- and metabolism-related genes, including the TCA cycle, consistent with reduced photosynthetic efficiency [79]. In contrast, RNA-seq analysis of plants infected with the endemic isolate TomCr3 showed the concurrent upregulation of photosynthesis, carbon fixation, and post-transcriptional gene silencing (PTGS) among the induced genes [83].

Despite similar viral titers, two isolates with varying levels of aggressiveness induced different transcriptomic responses in tomato plants upon PepMV infection [79]. Aggressive isolate triggered a stronger defense response mediated by SA signaling (including *PR1* and *PR5* induction) than JA-dependent pathways, thus indicating that PepMV aggressiveness is not correlated with the ability to suppress basal plant defenses. Hanssen et al. [79] also observed differential regulation of PTGS components with the induction of DCL2 (Dicer-like 2) and AGO2 (Argonaute 2), correlating with isolate aggressiveness. Tsitsekian et al. [83] expanded this framework by confirming that PTGS constitutes a major antiviral mechanism that positions RNA silencing as the principal axis of early control. Transcriptomic evidence from Alcaide et al. [80] further refined this view by revealing that different PepMV genotypes (EU and CH2) elicited distinct silencing signatures in both single and mixed infections. Key components of RNA silencing, namely *AGO1a*, *AGO2a*, *DCL2b*, and *DCL2d*, exhibit differential regulation dependent on both genotypes and temporal factors. In mixed infections (EU + CH2), the accumulation of CH2 was specifically reduced in the early stages, whereas the EU remained unaffected, thus indicating an asymmetric antagonism that decreased over time. Mechanistically, EU significantly upregulated *AGO2a* and differentially modulated *AGO1a*, *DCL2b*, and *DCL2d* at early stages, whereas CH2 did not affect *AGO2a*. The authors hypothesize that this difference could be due to the silencing suppressors (TGB1 and CP) of the CH2 strain being more efficient than those of the EU strain, thereby allowing CH2 to evade this host defense in single infections [80]. However, AGO2 is not the sole determinant of this antagonism, as evidenced by *ago2* mutant phenotypes in *N. benthamiana*, which confirms an important antiviral role, but does not eliminate the EU-on-CH2 effect [80]. The two genotypes also target distinct host pathways: CH2 preferentially affects lipid metabolism genes, potentially linked to replication complex membranes, whereas EU more strongly influences phytohormone-related and oxidative stress modules [80].

These transcriptional patterns align with genetic evidence: in *N. benthamiana*, AGO2 underpins a hierarchical PTGS cascade that shapes PepMV outcomes. For instance, *ago2* mutants exhibit severe stunting and accumulate up to five times more viral genomic RNA than the wild-type plants, and *AGO2* expression is robustly induced upon PepMV infection [84]. The strong activity of *AGO2* can mask the auxiliary antiviral roles of *AGO1A*, *AGO5*, and *AGO10*, which became evident only in *ago2* double mutants (e.g., *ago1a*/*ago2*, *ago2*/*ago5*, and *ago2*/*ago10*), all of which show significantly higher viral loads than *ago2* single mutants [84]. This layered defense is further structured by the vsiRNA source: AGO2 predominantly deploys primary vsiRNAs, whereas a largely independent RDR6-dependent pathway generates secondary vsiRNAs that are thought to be utilized by auxiliary AGOs to target the viral RNA. Consistently, *ago2*/*rdr6* double mutants display strong synergism with much more severe symptoms than either single mutant [84]. Collectively, these data highlight PTGS as a flexible and hierarchical antiviral barrier. The equilibrium between PepMV and its host is shaped by the sequential actions of multiple AGO proteins, which utilize different vsiRNA populations in a coordinated yet partially independent manner to counter the virus.

A compact transcription factor (TF) module appears to coordinate these shifts. Among the 84 differentially expressed host TFs identified by Tsitsekian et al. [83], 11 highly responsive regulators-including members of the NAC, bHLH, and C2H2 zinc-finger families-containing stress- and defense-related cis-elements such as ABA-responsive (ABRE) motifs in their promoters, consistent with hormone-redox integration. The C2H2 family exemplifies this regulatory polarity: a *ZAT1*-like gene (*Arabidopsis ZAT12* homolog) was upregulated, whereas *ZF2* (*ZAT10* homolog) was repressed, which suggests a division of labor between oxidative stress buffering and defense gene regulation. Meanwhile, repression of *Jasmonic Acid 2-Like* (*JA2L*), an NAC-family TF involved in stomatal behavior and JA signaling, indicates that PepMV may influence stomatal closure and modulate gas exchange and pathogen entry points through transcriptional control [83]. Intriguingly, several PepMV-responsive TFs share greater homology with rice than with their *Arabidopsis* counterparts, implying that these virus-responsive modules predate monocot-dicot divergence and represent an ancient transcriptional core for antiviral adaptation in angiosperms [83].

PepMV infection also disrupts secondary metabolism, particularly the carotenoid and phenylpropanoid pathways, which are crucial for fruit pigmentation and quality [79,83]. Genes encoding key enzymes such as phytoene synthase (*PSY*), ζ-carotene desaturase (*ZDS*), and lycopene β-cyclase (*LCYB*) are consistently downregulated, which leads to reduced pigment accumulation and the characteristic marbled fruit phenotype observed in infected tomatoes [79]. The suppression of carotenoid biosynthesis coincides with localized increases in phenolic and alkaloid defense metabolites, to underscore the metabolic reallocation from pigment production to stress-related secondary metabolism [79,83].

Collectively, these findings depict the early phase of PepMV infection as a period of profound and dynamic reprogramming that integrates metabolic repression, maladaptive defense activation, and transcriptional remodeling. PepMV acts as a sophisticated modulator of tomato physiology by reconfiguring the plant’s primary metabolism, hormonal balance, and gene-silencing machinery, while simultaneously altering fruit pigmentation and quality through interconnected molecular networks.

### 4.2. Environmental Modulation of Hormonal and Redox Crosstalk

Abiotic cues, such as salinity, reshape PepMV-tomato interactions by modulating the timing and amplitude of defense signaling and the coupling between transcriptional responses from viral accumulation [82]. Under non-saline conditions, PepMV robustly induces SA-responsive defense genes, most notably *PR1*, whereas both mild and high salinity attenuate this induction, indicating that salt stress congenotypes the SA pathway output [82]. In non-inoculated plants, salinity alone downregulated the majority of SA-, JA-, and ET-related transcripts, which confirms that ionic stress suppresses basal defense readiness [82]. A key finding was the lack of direct correlation between defense gene expression and viral load under stress [82]. Despite the dampened *PR1* induction under salt stress, PepMV genomic RNA accumulation was paradoxically reduced (6-fold) under mild salinity, while returning to control levels under high salinity. This non-linear relationship suggests that the expression level of defense-related genes does not directly correlate with viral accumulation. The authors hypothesized that this could be due to other factors, such as alterations in Ca^2+^ homeostasis or a “priming-like” effect under mild stress that leads to the buildup of virus-inhibiting compounds [82]. The expression of the JA-related marker *LoxD* and the ET-related marker *ACS1* showed an inverted pattern. Their RNA levels were significantly decreased under the low salinity and virus combination but recovered to levels comparable to the control group at high salinity. In contrast, the expression levels of canonical ABA markers *NCED1* and *ABA2* remained largely unchanged in virus-inoculated plants across all salinity levels, to indicate a selective rewiring of hormonal crosstalk rather than a universal stress response [82]. Overall, these findings highlight that plant responses to PepMV under salinity are non-linear and non-additive. This interaction reshapes defense gene expression in a manner that is independent of the resulting viral load and points to a complex regulatory network involving multiple signaling pathways and cellular factors.

Temperature also exerts a notable, genotype-dependent influence on PepMV accumulation and the extent of host reprogramming. Comparative analyses of thermo-tolerant (TT) and thermo-susceptible (TS) cultivars have revealed starkly opposite temperature optima for viral replication [81]. In TS plants, PepMV load was significantly reduced at moderate temperatures (26/20 °C) compared to extremely low (20/14 °C) or high (32/26 °C) temperatures. Conversely, TT plants exhibited the opposite pattern, with the viral load peaking at moderate temperatures, to suggest that constitutive heat tolerance traits reshape the antiviral capacity. This differential response is underpinned by extensive and distinct changes in the transcriptome. Notably, TT plants displayed a far more robust gene expression response to combined stress, with a significantly higher number of DEGs at temperature extremes compared to TS plants [81]. Pathway analysis further highlights contrasting strategies: TS plants maintained consistently upregulated plant–pathogen interaction pathways, whereas TT plants appeared to suppress these pathways at low temperatures and photosynthesis at high temperatures, possibly to reallocate resources for tolerance. In the TT background, glutathione transferase and aldehyde dehydrogenase upregulation at high temperatures coincided with lower viral loads, linking thermal cues to redox buffering and viral susceptibility. This finding aligns with previous study on SlGSTU38 (a tomato glutathione S-transferase), which interacts with PepMV CP [73]. In contrast, tonoplast intrinsic proteins (TIPs) were upregulated at low temperatures. Notably, the study also highlighted previously uncharacterized candidates, a DUF642-domain protein in TS and a DUF241-domain protein in TT, both downregulated under conditions favoring high viral accumulation, nominating them as putative modulators of temperature-dependent susceptibility [81]. In TS plants, the activation of mitogen-activated protein kinase (MPK3) at both temperature extremes illustrates how temperature stress can amplify defense signaling, yet this did not prevent high viral loads, which suggests a potential destabilization of the plant’s overall homeostasis when facing a combined viral threat [81].

Collectively, these findings indicate that environmental context, particularly salinity and temperature, acts as a higher-order regulator gating metabolic-defense integration, with redox-related genes such as SlGSTU38 mediating between abiotic and viral stress adaptation. PepMV-tomato interactions operate within an environmental framework in which genotype-specific thermotolerance and salt sensitivity affect defense activation.

### 4.3. Integrative Overview

Transcriptomic analyses revealed that PepMV induces complex host responses. Early infection suppresses photosynthesis and primary metabolism while activating redox and defense pathways. ROS-related enzymes and stress-responsive factors function as antiviral effectors but become susceptibility determinants when redox homeostasis fails. PepMV modulates PTGS components and m^6^A RNA methylation factors through viral suppressors. Hormonal signals and abiotic factors influence these responses. PepMV reprograms the tomato transcriptome through metabolic, redox, and RNA signaling. Infection outcome depends on host resilience versus viral manipulation. This plasticity explains the difficulty in achieving durable resistance.

## 5. Sources of Resistance and Breeding Challenges

PepMV poses a persistent challenge for tomato breeding due to its extensive genetic variability, frequent mixed infections, and rapid adaptability driven by mutation and recombination [7,8,15,65]. Most research efforts have focused on identifying resistance in wild *Solanum* species, as cultivated *S. lycopersicum* typically exhibits high susceptibility under experimental conditions [1,12].

### 5.1. Screening for Resistance in Wild Solanum Species

Extensive germplasm screening programs illustrate both the promise and the limitations of this strategy. Ling and Scott [85] examined a core collection of 109 accessions representing five *Solanum* species challenged with a mixture of PepMV-CH1 and PepMV-CH2 isolates. All 23 accessions of *S. lycopersicum* and 8 accessions of *S*. *pimpinellifolium* were fully susceptible, whereas moderate resistance occurred in *S*. *peruvianum* (LA0107, LA1305) and *S*. *chilense* (LA1971, LA2748). The most promising resistance was observed in three *S. habrochaites* accessions (LA1731, LA2156, and LA2167), where some plants remained symptomless. Progeny testing of LA1731 siblings showed resistance. When challenged with the Ch1/Ch2 mixture, all progeny plants were symptomless, though many were still infected. When challenged with TX1 (EU) isolate, the resistance was also evident [85]. Specifically, seven of the 10 inoculated LA1731-sib plants were asymptomatic, and six of those 10 tested negatives for PepMV TX1 via ELISA. This indicated that the resistance in this line restricts both symptom expression and viral accumulation, offering a source of broad-spectrum resistance.

Complementary work by Soler-Aleixandre et al. [86] on 229 accessions from 15 *Solanum* species found partial resistance against the EU-type isolate LE-2002 in several *S. chilense* accessions (ECU-527, LA0458, LA0470, LA1963, LA1968, LA1971, LA2762) and *S. peruvianum* accessions (CIAPAN-15, CIAPAN-16, PI212407, PI251311). In addition, *S. ochranthum* (ECU-335) exhibited mild symptoms and low viral titers, whereas *S. pseudocapsicum* (AN-CA-214) displayed complete resistance, with no detectable virus or visible symptoms. Furthermore, Soler et al. [87] screened 78 accessions from seven wild *Solanum* species against the EU-type isolate LE-2002 and identified *S. lycopersicoides* as another promising source of resistance. Four accessions (LA1964, LA4123, LA4126, and LA4131) produced 100% symptom-free plants with no detectable PepMV, whereas three additional accessions (LA1966, LA2408, and LA2951) showed only limited systemic infection and low virus accumulation. Partial resistance was also identified in *S. chilense*, where subsets of plants in four accessions (CDP03677, CDP04506, CDP06600, and LA1971) remained symptomless with low or undetectable titers [87]. Notably, the authors stressed that apparent resistance in leaves could mask uneven virus distribution and recommended multi-tissue diagnostics (apex, stem, roots) and sensitive assays to confirm the true absence of the virus. From a breeding standpoint, the strong resistance in *S. lycopersicoides*, together with the available introgression resources (e.g., lines derived from LA2951), positions it as a priority donor for transferring PepMV resistance into cultivated tomatoes [87].

A notable attempt to deploy wild *Solanum*-derived resistance in breeding programs was described in a 2017 patent [88], which reported the introgression of PepMV resistance from a single immune *S. peruvianum* plant (NCIMB 41927–42069) identified in a proprietary germplasm. Three quantitative trait loci (QTLs) associated with resistance were mapped on chromosomes 6, 7, and 9, and linked single-nucleotide polymorphism (SNP) markers were used for marker-assisted selection (MAS). Plants homozygous for all three QTLs exhibited high-level or near-complete resistance to PepMV, which was effective against the EU, CH2, and LP genotypes. However, early backcross generations showed reduced fruit set and vigor, which indicated a linkage drag from *S. peruvianum*-derived chromosomal regions [88]. Further backcrossing improved agronomic traits but did not fully stabilize the resistance under greenhouse conditions. To our knowledge, no widely adopted commercial cultivar derived from this material has been documented, which may reflect unresolved challenges in trait stability or yield performance in variable environments.

Preliminary observations from the authors’ laboratory further illustrate these complexities. When challenged with the aggressive necrotic isolate PepMV-P19 (CH2), *S. habrochaites* LA2167 was fully susceptible, whereas LA1731 remained symptomless but was virus-positive by RT-qPCR [89]. This pattern corresponds to tolerance, the suppression of visible symptoms despite active viral replication, rather than complete resistance.

Although these sources show promise, their use in breeding is limited by the presence of reproductive barriers (Table 1). *S. pseudocapsicum* is completely incompatible with cultivated tomatoes. Hybrids with *S. chilense* can be produced, but they often require embryo rescue [90]. Similarly, *S. lycopersicoides* is distantly related to *S. lycopersicum*, which complicates interspecific hybridization. However, in the case of accession LA2951, pre-developed introgression lines in the tomato background provide a framework for the transfer of resistance alleles [91]. Even in partially compatible crosses, linkage drag and hybrid sterility often hinder backcross recovery. Consequently, introducing PepMV resistance from wild relatives is difficult and frequently requires bridge crosses, embryo rescue, or somatic hybridization to overcome both pre- and post-zygotic barriers [85,86,87,90].

### 5.2. Limitations and Perspectives for Durable Resistance

Developing durable resistance to PepMV remains a complex challenge. As discussed in Section 2.5, canonical resistance genes such as *Rx*, which are effective against other potexviruses, fail to provide stable protection against PepMV because of its capacity to evade recognition through minor changes in the CP [58]. These limitations reflect the broader trends observed with rapidly evolving RNA viruses and underscore the necessity for alternative approaches [92]. As noted in Section 3.5, resistance identified via classical mutagenesis, such as the *slosca4.1* mutant line 2F531, relies on loss of susceptibility (LOS) rather than immune recognition [78]. Although this strategy offers broader-spectrum protection, it still requires validation across viral genotypes and mixed infection scenarios.

The limitations of both natural and induced resistance highlight the need for alternative strategies that extend beyond the conventional gene-for-gene approaches. Emerging tools in functional genomics and plant biotechnology, particularly CRISPR-based gene editing and RNA silencing technologies, provide new opportunities to target viral susceptibility factors or trigger sequence-specific antiviral responses [93,94,95,96]. These strategies are discussed in greater detail in Section 7, where their potential for achieving precise and durable resistance is evaluated. However, their implementation is subject to regulatory constraints and practical deployment challenges, especially within the European Union, where current frameworks still treat genome-edited crops as genetically modified organisms (GMOs) [97,98].

Ultimately, overcoming the limitations of traditional resistance breeding will require a combined approach that integrates novel biotechnological tools with careful pathogen monitoring, environmental assessment, and breeding strategies designed for long-term stability and commercial scalability.

## 6. Current Control Methods

The management of PepMV in greenhouse tomato production relies primarily on preventive hygiene and biological protection, as no broadly adopted cultivars with stable genetic resistance are yet available [1,12]. Accordingly, strict hygiene and seed sanitation form the operational core of the control programs (see Section 2.1 for transmission biology). Disinfection of seeds with both chemical (e.g., 0.5–1.0% sodium hypochlorite or 10% trisodium phosphate) or controlled heat treatments can markedly reduce infectivity without impairing germination and is now a routine element of integrated management [1,31,99].

Among the biological methods, cross-protection remains the most effective strategy for managing PepMV infections [10,12]. This relies on pre-inoculating tomato plants with a mild viral isolate that prevents or mitigates infection by more aggressive genotypes of the virus. The commercial use of this approach, initiated more than two decades ago, has proven highly effective in reducing fruit marbling and yield losses [10,11,12,100]. Mild isolates from both the EU and CH2 genotypes (e.g., CH2-1906, VX1/VC1, and PMV^®^-01) are authorized in Europe as low-risk biological control agents [101,102]. Large-scale implementation, particularly in Spain, has demonstrated substantial economic benefits, estimated at several hundred thousand tons of preserved tomato yield annually [12]. Experiments have shown that mixtures of mild isolates PepMV-Sp13 (EU genotype) and PepMV-PS5 (CH2 genotype) provide broad protection against aggressive isolates from both genotypes. No recombinant viruses were detected after multiple passages, and mixed infections remained mild and stable across the cultivars and conditions [4]. These findings validate the safe use of mixed mild-isolate formulations in commercial practices.

The mechanisms underlying cross-protection are based on two main hypotheses. The first is RNA silencing (RNAi), where infection with a mild isolate induces the production of virus-derived small RNAs (siRNAs) that recognize and degrade the RNA of a subsequent homologous, more aggressive virus [12]. The second hypothesis, superinfection exclusion (SIE), proposes that the initial mild virus engages essential host resources such as replication sites, making them unavailable to the challenging severe virus [103]. Experiments with *N. benthamiana* mutants deficient in key RNA silencing components (AGO2 and DCL2/4) showed that pre-inoculation with a mild PepMV isolate still conferred complete protection against a severe isolate [103]. These findings suggest that the protective mechanism is likely distinct from classical RNA silencing and more aligned with the SIE model [103]; however, further validation is necessary before applying them to the tomato–PepMV system.

Ultimately, the efficacy and biosafety of cross-protection critically depend on adherence to the manufacturer’s recommendations, including inoculation timing, dosage, and hygiene standards. Deviations from these guidelines, which are frequent in some production systems, can compromise protection and favor unintended viral spread. When properly implemented and monitored, cross-protection is the only field-validated, biologically based strategy that consistently reduces the impact of PepMV in commercial tomato cultivation.

Some complementary, still-experimental options include small-molecule antivirals and elicitors. Foliar application of three 1,3,4-oxadiazole derivatives (OH-Oxa, CH_3_-Oxa, NO_2_-Oxa) and of the derivative of 1,3,4-thiadiazole (OH-Thia) reduced PepMV accumulation and symptom severity in tomato leaves [104]. Similarly, Ezzouine et al. [105] demonstrated that treatments with chitosan and chitosan–nano-hydroxyapatite significantly reduced PepMV infection in tomato plants. In addition to restricting virus spread, these treatments conferred protection against *Verticillium dahliae*, thereby highlighting their dual protective effect [105]. The ability of chitosan-based treatments to induce systemic resistance further supports their potential for sustainable PepMV management.

In practice, the current integrated management combines preventive hygiene, seed sanitation, and biological protection through cross-protection, with elicitors and antivirals considered where appropriate. The proven performance of cross-protection in commercial settings underscores its importance. At the same time, its reliance on live viral inocula and sensitivity to PepMV population structure highlights the need for continuous molecular monitoring and careful stewardship. Advances in chemical elicitors, antiviral agents, and ecologically informed IPM designs are expected to strengthen the robustness and sustainability of the integrated PepMV control strategies going forward.

## 7. Emerging Strategies and Durable Resistance for PepMV in Tomato

The lack of durable resistance to PepMV remains a major constraint in tomato breeding programs. To the best of our knowledge, no commercial cultivar with stable resistance has been developed despite nearly two decades of intensive efforts, including the exploration of wild *Solanum* relatives and QTL introgressions. The genetic complexity and frequent recombination rates of the virus have repeatedly undermined classical resistance approaches, thus making PepMV one of the most challenging pathogens in tomato cultivation. As a result, management relies mainly on cross-protection with mild PepMV isolates, which substantially reduces symptom severity and yield loss. However, this method does not eliminate the virus and carries the risk of recombination or reversion to virulence.

### 7.1. Lessons from Other Tomato Viruses

Progress against other major tomato viruses has demonstrated that durable genetic resistance is achievable in some pathosystems when viral evolution is effectively constrained. The introgression of the *Ty* gene series (e.g., *Ty-1*, *Ty-2*, and *Ty-3*) from wild relatives has provided long-lasting control of the tomato yellow leaf curl virus (species *Begomovirus coheni*, TYLCV) in many regions [106,107,108]. Tolerance and resistance to ToBRFV have recently been reported in wild *Solanum* species, thereby offering promising breeding materials [109,110,111,112,113]. In recent years, seed companies have announced cultivars described as resistant or tolerant to ToBRFV [114,115]. These examples illustrate that durable control is feasible in specific virus–host systems, particularly when the targeted functions show limited evolutionary lability. In contrast, PepMV remains more challenging, reflecting its genetic complexity and the multifunctionality of several viral proteins. Accordingly, for PepMV, it is prudent to prioritize the evaluation of host-centered strategies alongside conventional approaches, while continuing to benchmark their durability.

### 7.2. Classical Mutagenesis and Targeted Genome Editing Approaches

Plant RNA viruses possess compact genomes and consequently depend on host factors, referred to as susceptibility factors, to complete their life cycles [95]. Accordingly, mutating or silencing a host factor gene can confer LOS resistance, which is typically achieved via targeted genome editing (e.g., CRISPR/Cas) or gene-silencing approaches [95,96]. This concept has been extensively validated, as demonstrated by CRISPR-derived tomato quadruple *tom1* mutants that exhibited high resistance, including to ToBRFV, without any growth penalties [116]. Beyond targeted editing, random mutagenesis with chemicals (e.g., EMS) and physical agents (gamma rays) remains a powerful discovery route, as it generates diverse genome-wide allelic series without transgenes [117], whereas TILLING provides a reverse genetics screen to identify individuals carrying mutations in predefined host factor loci [118,119]. This pipeline has proven to be efficient and cost-effective and has yielded *eif4e* mutants resistant to two potyviruses (PVY and PepMoV) in tomato [120].

Within this broader context, PepMV research has validated both random and targeted approaches to LOS resistance. Chemical mutagenesis identified the tomato line 2F531, which carries a mutation in *SlOSCA4.1* that confers resistance to diverse PepMV genotypes without affecting plant growth [78]. Similarly, genome editing of *SlGSTU38* impairs viral accumulation by altering redox homeostasis [73]. These studies highlight the feasibility of disabling the host factors that are critical for PepMV replication. A combined strategy integrating classical mutagenesis, genome editing, and RNA-based suppression may provide a multilayered defense that is less prone to resistance breakdown [93,94,121,122].

### 7.3. RNA-Based Strategies: Transgenic and Non-Transgenic

Biotechnological progress has opened new possibilities for PepMV control through RNA silencing (RNAi), which operates via two complementary mechanisms: host-induced gene silencing (HIGS) and spray-induced gene silencing (SIGS). In HIGS, transgenic tomato plants express viral hairpin constructs that trigger the accumulation of small interfering RNAs (siRNAs) to prevent viral replication. Two tomato lines, 431 and 432, expressing an inverted-repeat construct (IR-PepMV) derived from the conserved regions of three distinct PepMV genotypes (US1, LP, and CH2), showed complete immunity to a broad spectrum of isolates. This included aggressive variants such as EU-a, LP-m, US1-a, CH2-a, CH2-m, CH2-pvu, and CH2-IL [122]. These plants maintained full protection for at least three months under simulated commercial growth conditions, thus confirming the durability of the transgene-derived siRNA (tr-siRNA) approach. However, grafting experiments demonstrated that this protection was confined to transgenic tissues, as siRNAs produced in the rootstock failed to confer resistance to non-transgenic scions, underscoring the need for whole-plant transformation [122]. Similar RNAi-based constructs have been proven effective against other major tomato viruses, including TYLCV [123,124] and TSWV [125]. Despite their efficacy, HIGS approaches remain limited by strict regulatory frameworks and persistent public concerns regarding genetically modified crops.

SIGS presents a non-transgenic alternative by applying exogenous double-stranded RNAs (dsRNAs) that directly target viral genes in plants, thereby triggering endogenous RNAi and suppressing viral replication [126,127]. This approach combines high sequence specificity with minimal off-target risk and an environmentally friendly profile, and shows promising results in other virus–crop systems [128]. For example, it has effectively delayed the appearance of TSWV symptoms [129] and offers a promising method for controlling CGMMV (Cucumber green mottle mosaic virus) in cucumbers [130], although its performance still depends on the species, formulation, and environmental conditions [128]. Advances in delivery and stability, such as nanoparticle encapsulation, layered double-hydroxide (LDH) clay nanosheets, and biodegradable polymers, have enhanced foliar persistence and dsRNA uptake [127]. SIGS is still in its early stages and requires thorough greenhouse and field validation, formulation optimization, and safety assessment. Moreover, no PepMV-specific SIGS data are currently available. However, the technique’s precision, flexibility, and sustainability make it a promising, forward-looking addition to integrated virus management once evidence specific to PepMV becomes available.

### 7.4. Regulatory and Practical Considerations

The deployment of biotechnological solutions for PepMV control through genome editing or HIGS depends on the regulatory frameworks governing the GMOs. Within the European Union, genome-edited and transgenic plants are regulated as GMOs under Directive 2001/18/EC regardless of the presence of foreign DNA. Even minimal transgene-free edits require multi-year authorization procedures, environmental risk assessments, and field-trial permits, thus delaying commercialization [97,131]. Field trials require detailed dossiers and restrictive containment conditions, slowing the transition of innovations such as *slosca4.1* mutants to agricultural settings. Product-based regulatory systems in countries such as the United States, Japan, Argentina, and Australia evaluate the end product rather than the production method [132,133]. Under these frameworks, genome-edited plants without transgenes are generally exempt from GMO regulations, which enables faster translation into breeding programs. Tomato lines with intragenic RNAi constructs targeting CMV and TSWV were classified as non-GMOs by the USDA-APHIS, thus facilitating rapid market entry. The same material remains a GMO under EU law [98,134]. This regulatory asymmetry means that PepMV-resistant cultivars obtained through HIGS or genome editing will likely be commercialized earlier outside Europe. The proposed EU reforms aim to introduce a tiered system that distinguishes targeted mutagenesis and cisgenic or intragenic plants from conventional transgenic plants. Such differentiation could facilitate the deployment of genome-edited and RNAi-based resistance strategies in European breeding programs. Although regulatory hurdles remain substantial, global trends indicate the growing acceptance of precision, non-transgenic biotechnology for plant virus management. Aligning regulations with scientific advances is essential for translating molecular innovations into practical solutions for PepMV control. Beyond technical feasibility, adoption will depend on regulatory harmonization and socio-economic factors, including cost, consumer acceptance, and intellectual property frameworks.

### 7.5. Outlook and Knowledge Gaps

**Figure 3:**
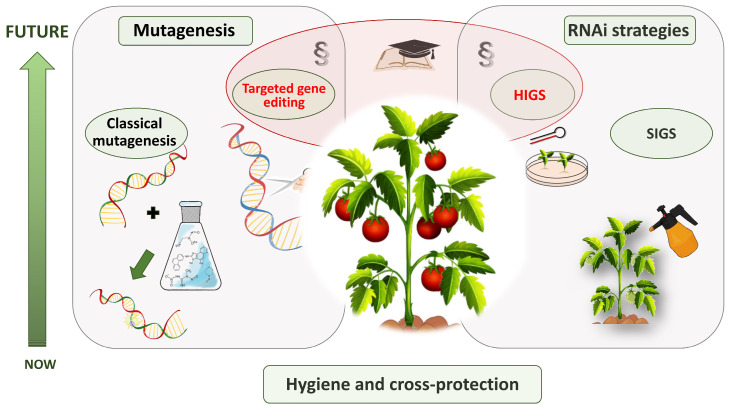
Conceptual roadmap for PepMV management in greenhouse tomato. The vertical arrow represents the temporal progression from current strategies (NOW) to future approaches (FUTURE). The central circle highlights the tomato host. **Left**: mutagenesis (e.g., classical EMS/TILLING; CRISPR/Cas gene editing). **Right**: RNAi strategies, including HIGS (transgenic) and SIGS (non-transgenic). The gavel/§ icons indicate stages likely requiring regulatory approval. These tools are complements to hygiene and cross-protection and should be coupled with genotype-resolved diagnostics and surveillance.

PepMV is one of the most adaptable and economically pervasive viral threats to tomato cultivation. However, recent advancements in mutagenesis, genome editing, and RNA-based control have revolutionized the conceptualization and implementation of resistance strategies (Figure 3). The successful development of PepMV-resistant cultivars will ultimately rely on molecular innovation, regulatory harmonization, economic feasibility, and social acceptance. Integrated management, rooted in hygiene, surveillance, and controlled biological protection, continues to be the cornerstone of PepMV control, whereas molecular breeding lays the groundwork for the next generation of durable, sustainable resistance.

Despite rapid advances in PepMV epidemiology, molecular characterization, and host resistance strategies, several critical gaps remain [5,9,48,73,78]. Key unresolved questions include: (1) the mechanisms underpinning durable genetic resistance in tomato, (2) the evolutionary dynamics of new emerging genotypes and recombinants of PepMV, and (3) the functional interplay of host susceptibility factors during infection cycles. Technologically, urgent priorities include: large-scale discovery and functional validation of resistance genes, application of genome editing to engineer loss-of-susceptibility and durable resistance without yield penalties, and the development of sensitive molecular diagnostics capable of discriminating emerging PepMV genotypes.

Collaborative multidisciplinary efforts linking virologists, breeders, molecular biologists, and epidemiologists, are vital. Future work should prioritize: (1) leveraging advanced genomics and transcriptomics for resistance breeding, (2) integrating biological and chemical controls in a systems approach, and (3) navigating rapid deployment and regulatory adaptation for editing technologies across regions.

## 8. Conclusions

PepMV poses a continued major threat to tomato production worldwide due to its unique biological and molecular characteristics. The ability of the virus to spread through mechanical contact, coupled with a relatively high substitution rate and recombination potential, enables it to rapidly adapt to new environments and host defenses. Furthermore, the capacity of PepMV to coexist with other viruses in mixed infections adds another layer of complexity to its management. These characteristics collectively contribute to the persistence of the virus and the challenges faced in developing resistant cultivars.

Although current strategies, such as cross-protection, have shown promise in mitigating disease severity, they fail to address the core issue of genetic resistance in tomato plants. This gap has spurred research into innovative approaches, particularly in biotechnology. Genome editing techniques, such as CRISPR/Cas9, offer the potential to modify plant genes to confer resistance without the need for traditional breeding methods. Similarly, RNAi strategies can be employed to silence viral genes, to potentially provide a more targeted and efficient means of control. However, the successful implementation of these technologies requires a comprehensive understanding of PepMV epidemiology and evolution. Integrating advanced biotechnological tools with the ongoing surveillance of viral populations and careful management of environmental factors is crucial for developing sustainable, long-term solutions to protect tomato crops from the persistent threat of PepMV. The integration of genome editing and SIGS, coupled with regulatory reforms and cost-effective deployment, represents a promising frontier for durable PepMV resistance.

## Figures and Tables

**Table 1:** Documented sources of PepMV resistance in wild *Solanum* germplasm.

Species	Accession(s)	Resistance Level	Crossability with *S. lycopersicum*	Reference
*S. lycopersicoides*	LA1964, LA4123, LA4126, LA4131	Complete resistance (symptom-free, no virus detected)	Highly incompatible (introgression lines required)	[87]
	LA1966, LA2408, LA2951	Moderate resistance (limited systemic infection, reduced virus accumulation)		[87]
*S. pseudocapsicum*	AN-CA-214	Complete resistance (symptom-free, no virus detected)	Incompatible	[86]
*S. habrochaites*	LA1731	Broad spectrum resistance (symptom-free or reduced symptoms, virus detectable)	Partially compatible (interspecific hybrids often show reduced fertility)	[85], this study
	LA2156, LA2167	Variable resistance (symptom suppression or susceptible)		[85], this study
*S. peruvianum*	CIAPAN-15, CIAPAN-16, PI212407, PI251311, LA0107, LA1305	Moderate resistance (reduced virus accumulation)	Partially compatible (embryo rescue often required)	[85,86]
	NCIMB 41927–42069	High to near-complete resistance		[88]
*S. chilense*	ECU-527, LA0458, LA0470, LA1963, LA1968, LA1971, LA2762, CDP03677, CDP04506, CDP06600, LA2748	Moderate resistance (subsets symptom-free; low or undetectable virus accumulation)	Partially compatible (frequent embryo rescue)	[85,86,87]
*S. ochranthum*	ECU-335	Moderate resistance (mild symptoms; reduced virus accumulation)	Highly incompatible	[86]

## Data Availability

No new data were created or analyzed in this study. Data sharing is not applicable to this article.

## Abbreviations

The following abbreviations are used in this manuscript:

APHIS	Animal and Plant Health Inspection Service
CAS	CRISPR-associated system
CH2	Chilean 2 genotype (of PepMV)
CMV	Cucumber mosaic virus
CP	Coat protein
CRISPR	Clustered regularly interspaced short palindromic repeats
DNA	Deoxyribonucleic acid
dsRNA	Double-stranded RNA
EAP	Europe–Asia–Pacific lineage (of PepMV)
ECT	Evolutionarily Conserved C-Terminal region
ELISA	Enzyme-linked immunosorbent assay
EMS	Ethyl methanesulfonate
EU	European genotype (of PepMV)
GMO	Genetically modified organism
HIGS	Host-induced gene silencing
LDH	Layered double hydroxide
LOS	Loss of susceptibility
LP	Peruvian-like genotype (of PepMV)
MAS	Marker-assisted selection
PepMoV	Pepper mottle virus
PepMV	Pepino mosaic virus
PES	Southern Peruvian genotype (of PepMV)
PVY	Potato virus Y
QTL	Quantitative trait locus
RDR	Host-encoded RNA-dependent RNA polymerase
RNA	Ribonucleic acid
RNAi	RNA interference
RT-qPCR	Reverse transcription quantitative polymerase chain reaction
SIGS	Spray-induced gene silencing
siRNA	Small interfering RNA
SNP	Single-nucleotide polymorphism
ToBRFV	Tomato brown rugose fruit virus
TSWV	Tomato spotted wilt virus
TILLING	Targeting induced local lesions in genomes
TYLCV	Tomato yellow leaf curl virus
US1	North American genotype (of PepMV)
